# Cholinergic Syndrome: a Case Report of Acute Organophosphate and Carbamate Poisoning

**DOI:** 10.2478/aiht-2020-71-3413

**Published:** 2020-06-29

**Authors:** Tadej Petreski, Barbara Kit, Matej Strnad, Damjan Grenc, Franc Svenšek

**Affiliations:** 1 University Medical Centre Maribor, Clinic for Internal Medicine, Department of Nephrology, Maribor, Slovenia; 2 University of Maribor, Faculty of Medicine, Maribor, Slovenia; 3 University Medical Centre Maribor, Clinic for Internal Medicine, Intensive Care Unit, Maribor, Slovenia; 4 Centre for Emergency Medicine, Prehospital Unit, Maribor, Slovenia; 5 University Medical Centre Ljubljana, Clinic for Internal Medicine, Centre for Clinical Toxicology and Pharmacology, Ljubljana, Slovenia

**Keywords:** carbofuran, chlormephos, critical care, obidoxime, karbofuran, klormefos, intenzivna medicina, obidoksim

## Abstract

Cholinergic syndrome is a common topic at western medical universities yet rarely observed in clinical practice. The treatment involves muscarinic antagonists, acetylcholinesterase reactivation, seizure control, and supportive measures. Here we report a case of a 52-year old Caucasian male who attempted suicide by ingesting a purple crystal powder that turned out to be a mixture of carbofuran and chlormephos. At clinical examination, the patient presented with salivation, perspiration, diarrhoea, bradypnoea, loss of consciousness, and epileptic seizures. Laboratory tests showed low plasma cholinesterase, and we started obidoxime along with supportive intensive care treatment. He was later transferred to the psychiatry department for further diagnostics and treatment.

## Abstract (hr)

Holinergični sindrom je pogosta tema na medicinskih fakultetah v zahodnem svetu, ki pa ga redko obravnavamo v klinični praksi. Zdravljenje sestoji iz uporabe muskarinskih antagonistov, reaktivacije acetilholinesteraze, nadzora nad epileptičnimi napadi in podpornih ukrepov. Predstavljamo primer 52-letnega moškega, ki je poskušal storiti samomor z zaužitjem vijoličnega kristalnega prahu, ki se je izkazal za mešanico karbofurana in klormefosa. Ob pregledu smo opažali povečano slinjenje in potenje ter prisotnost driske, bradipneje, izgube zavesti in epileptičnih napadov. V laboratorijskih preiskavah smo beležili nizke vrednosti plazemske holinesteraze in pričeli zdravljenje z obidoksimom ob podpornem intenzivnem zdravljenju. Kasneje smo ga premestili na Oddelek za psihiatrijo za nadaljnjo diagnostiko in zdravljenje.

Organophosphates (OPs) are pesticides that inhibit acetylcholinesterase (AChE) in synapses with irreversible binding, which causes accumulation of acetylcholine (ACh) and overstimulation of nicotinic and muscarinic cholinergic receptors (1, 2, 3). This can be measured with the erythrocyte ACh assay, which is not readily available in all laboratories. An alternative marker for diagnosis is plasma cholinesterase (ChE), also known as butyrylcholinesterase (BuChE), which is produced by the liver and has a drawback of not accurately reflecting the severity of poisoning (1, 4). It circulates in the blood and does not have any known physiological function (5). Carbamates are similar compounds that cause a transient AChE inhibition, which lasts about 48 h (2).

OPs can cause cholinergic toxidrome, intermediate syndrome, OP-induced delayed polyneuropathy, and chronic OP-induced neuropsychiatric disorder (5). This case report will focus mainly on acute manifestations of cholinergic syndrome. Severe muscarinic cholinergic symptoms are easily remembered from textbooks by two mnemonics: SLUDGE/BBB (salivation, lacrimation, urination, diaphoresis, gastro-intestinal upset, emesis, bronchorrhoea, bronchospasm, bradycardia) and/or DUMBBBELS (diarrhoea, urination, myosis, bronchorrhoea/bronchospasm/bradycardia, emesis, lacrimation, salivation). Additionally, an exposed person might experience fasciculation, muscle weakness, paralysis, acute lung injury, central nervous system (CNS) depression, agitation, confusion, seizures, and coma (1, 2, 5, 6). Hypotension, ventricular dysrhythmias, metabolic acidosis, pancreatitis, and hyperglycaemia may also develop. As acute effects of OP poisoning are well known, several recent studies have shifted focus on its chronic effects and additional OP and carbamate targets such as oxidative stress, axonal transport deficits, neuroinflammation, and autoimmunity, the knowledge of which could provide new treatment possibilities (7, 8).

Treatment of acute poisoning usually involves resuscitation, decontamination, symptomatic and supportive treatment, including oxygen supplementation, extracorporeal cardiopulmonary support, and early seizure control. Specific antidotes used in OP poisoning are muscarinic antagonists (e. g. atropine) and oximes as AChE reactivators (1, 4, 6), If hospital supplies of atropine are limited or CNS anticholinergic toxicity is present before adequate symptom control is achieved, glycopyrrolate can be given, and if respiratory symptoms are the main problem, ipratropium inhalations can also be administered (6). The use of antioxidants is still a controversy, as some animal and human studies report no substantial benefits against OP poisoning, while others do for several substances (7, 8, 9, 10).

## Case report

A 52-year old Caucasian male, previously treated for alcohol abuse, otherwise healthy, was admitted to the intensive care unit due to bradypnoea. He was found having an epileptic seizure by a local doctor, who administered 10 mg of diazepam, which stopped the seizures. When the emergency doctor arrived, the patient was unconscious, bradypnoic (6 breaths/min), and cyanotic (SpO_2_ without supplemental oxygen at 60 %). He underwent rapid sequence intubation with etomidate, rocuronium, fentanyl, and midazolam. During transport to the hospital the patient had narrow pupils and salivated excessively. In the emergency department he underwent head CT and chest X-ray, which did not detect abnormalities.

Upon admission to the intensive care unit (ICU), approximately 2 h after the seizure, we additionally observed excessive perspiration and diarrhoea but no other signs of excessive muscarinic stimulation such as urinary incontinence, nausea, vomiting, bronchospasm, or bronchorrhoea). The patient was haemodynamically stabilised after fluid resuscitation and did not need any vasopressor or inotropic support.

Initial laboratory tests revealed leucocytosis (17.5/mm^3^), hyperglycaemia (14 mmol/L), high creatine kinase (10.29 μkat/L), hyperlactataemia (3.0 mmol/L), and hypokalaemia (2.96 mmol/L). The patient was acidotic (pH 7.08), with bicarbonate level of 23.7 mmol/L, pCO_2_ of 11.2 kPa, and pO_2_ of 15.5 kPa. In addition, serum ChE was low (32 μkat/L).

Basic urine tox screen for opiates, amphetamines, cocaine, cannabis, and methadone was negative and so was serum alcohol. Table 1 shows relevant laboratory findings and how they changed over the first three days at ICU.

**Table 1:** Timeline of pathological laboratory findings in our patient with acute OP and carbamate poisioning

	Day 1		Day 2	Day 3	Reference values
Hour of measurement (hh:mm)	**11:40**	**17:50**	**7:50**	**8:00**	
ChE (μkat/L)	32	30	31	140	117–317
Lactate (mmol/L)	3.0	4.2	1.5	2.2	0.5–1.8
CRP (mg/L)	<3		46	111	0–5
Leucocytes (/mm3)	17.47		12.64	8.95	4–10
Potassium (mmol/L)	2.96	3.18	4.38	3.93	3.5–5.3
CK (μkat/L)	10.29		4.04		0–2.42

ChE – cholinesterase; CRP – C-reactive protein; CK – creatine kinase

The patient was treated empirically with amoxicillin and clavulanic acid for possible aspiration pneumonia. *Serratia marcescens*, *Escherichia coli*, and *Haemophilus influenzae* were isolated from tracheal aspiration, and the antibiotic was continued following the antibiogram. Due to suspected poisoning with cholinergic substances, we consulted the national Poison Control Centre in Ljubljana, Slovenia and sent urine and gastric lavage fluid samples to the Institute for Forensic Medicine, Ljubljana, where they identified carbofuran in urine and a combination of carbofuran and chlormephos in the gastric fluid. In addition, the patient’s relatives brought a bag of purple crystal powder they found at home next to ground coffee, which contained the same substances found in the gastric fluid. They did not know how much was ingested but thought that he could have drunk it with his morning coffee.

We began treatment with obidoxime intravenously (iv) approximately 10 h after admission. Initially, we administered a 250 mg bolus, which was followed by a continuous infusion of 750 mg diluted in 500 mL of 0.9 % sodium chloride (NaCl) solution that lasted 24 h. The patient continued to receive standard intensive care medication: analgesics, sedatives, prophylactic low-molecular weight heparin, proton pump inhibitors, and balanced iv fluids. He also received thiamine parenterally due to history of alcohol abuse.

In the first eight hours at ICU, he experienced additional short-term epileptic seizures, which were managed with lorazepam. We do not believe the patient has had any other indications or conditions that could provoke seizures other than poisoning. No further seizures developed after administration of obidoxime. EEG showed abnormal readings due to badly defined, irregular, and distinctly slowed baseline activity. It also showed some bursts of slow delta waves over the frontal, frontotemporal, and frontocentral brain regions. A neurologist was consulted, who concluded that the seizures were due to metabolic causes, so no further imaging was done.

After five days, a psychiatrist was consulted, who reported symptoms of depression and possibility of suicide attempt, which the patient denied at the time. Haemodynamically and respiratory stable, he was transferred to the psychiatry ward for observation and additional diagnostics. Some time after his transfer to psychiatry, he reported that he intentionally drank some 30-year-old poison, because his wife left him a month earlier and he wanted to die.

## Discussion

The substances found in the powder, gastric lavage, and urine were chlormephos and carbofuran. Chlormephos is an organothiophosphate soil insecticide, while carbofuran is a carbamate insecticide, acaricide, and nematicide (11, 12).

Data on chlormephos toxicity are scarce. Its molecular formula is C_5_H_12_ClO_2_PS_2_. It usually takes the form of a colourless liquid that is stable in neutral and weakly acidic media at room temperature and solves very poorly in water. Following oral administration in rats, it is almost completely eliminated by the kidney within 24 h. Absorption and effects are believed to be similar to other OP compounds. Its oral median lethal dose (LD_50_) in rats is high, 7 mg/kg (12). Carbofuran, on the other hand, is well known, and has an even higher LD_50_ of <50 mg/kg (6). Both substances are quite rare in Slovenia now, but individuals might still keep their products bought many years ago.

Benzodiazepines should be administered in cholinergic syndrome for seizure control as soon as possible, as they tend to lose effectiveness with delay of treatment due to rapid desensitisation of synaptic GABA A receptors (6, 13). If this happens, synthetic neurosteroids such as alphaxolone, minaxolone, and ganaxolone may serve as alternative. They are currently being studied in preclinical trials on rats because of stronger anticonvulsant effects that are not limited by GABA desensitisation (13). Atropine and oxime therapy were introduced into clinical practice in the 1950s. The main idea of using antimuscarinic therapy (atropine) is to maintain the heart frequency between 80 and 100 beats per minute, raise systolic blood pressure above 80 mmHg, and clear bronchial obstruction. Atropine should be started with 1–2 mg boluses, and the dose doubled every 5 min until the targeted clinical response is achieved. A continuous infusion should follow with 10–20 % of the total dose administered per hour. Resuscitation fluid should also be administered, and when haemodynamic instability persists catecholamines are often indicated (1, 3, 4, 6). As our patient was not bradycardic and did not show any other signs of excessive muscarinic stimulation save for perspiration and diarrhoea, we decided against atropine therapy. His blood pressure was well controlled with fluid resuscitation and he received bronchodilation therapy with inhalations during mechanical ventilation.

The use of AChE reactivators is crucial in organophosphate poisoning as atropine is not effective in the neuromuscular synapse. Obidoxime should be started as a 250 mg iv bolus over 20–30 minutes. Continuous infusion of 30 mg/h should follow for 12–24 h, that is, until atropine is not needed (if used) and the patient has been extubated (1, 3). However, one should be cautious when using oximes in carbamate poisoning. Studying the effects of oximes against carbamate toxicity *in vitro* Wille et al. (14) observed that AChE inhibition rates were higher when they used an oxime against carbamates carbaryl or propoxur, but not when they used it against carbofuran. As we did not use atropine, we limited the treatment to 24 h. The patient’s condition was continuously improving after the initiation of oxime therapy, and we observed no adverse reactions. One day after we stopped oxime treatment, we observed a rise in plasma ChE and the patient was gradually extubated as clinical and laboratory parameters improved.

In conclusion, even though carbamates and OPs are now rare due to stricter regulations, presentation with cholinergic symptoms should immediately raise suspicion of OP or carbamate poisoning and prompt identification of the substance used and corresponding treatment, as individuals still keep these products stored at home.
